# Combining thermal imaging and soil water content sensors to assess tree water status in pear trees

**DOI:** 10.3389/fpls.2023.1197437

**Published:** 2023-06-06

**Authors:** Victor Blanco, Noah Willsea, Thiago Campbell, Orlando Howe, Lee Kalcsits

**Affiliations:** ^1^ Tree Fruit Research and Extension Center, Washington State University, Wenatchee, WA, United States; ^2^ Department of Horticulture, Washington State University, Pullman, WA, United States

**Keywords:** ‘Bartlett’, CWSI, ‘d’Anjou’, thermal-based indices, stem water potential, soil moisture, stomatal conductance, ‘Williams’

## Abstract

Volumetric soil water content is commonly used for irrigation management in fruit trees. By integrating direct information on tree water status into measurements of soil water content, we can improve detection of water stress and irrigation scheduling. Thermal-based indicators can be an alternative to traditional measurements of midday stem water potential and stomatal conductance for irrigation management of pear trees (*Pyrus communis* L.). These indicators are easy, quick, and cost-effective. The soil and tree water status of two cultivars of pear trees ‘D’Anjou’ and ‘Bartlett’ submitted to regulated deficit irrigation was measured regularly in a pear orchard in Rock Island, WA (USA) for two seasons, 2021 and 2022. These assessments were compared to the canopy temperature (Tc), the difference between the canopy and air temperature (Tc-Ta) and the crop water stress index (CWSI). Trees under deficit irrigation had lower midday stem water potential and stomatal conductance but higher Tc, Tc-Ta, and CWSI. Tc was not a robust method to assess tree water status since it was strongly related to air temperature (R = 0.99). However, Tc-Ta and CWSI were greater than 0°C or 0.5, respectively, and were less dependent on the environmental conditions when trees were under water deficits (midday stem water potential values< -1.2 MPa). Moreover, values of Tc-Ta = 2°C and CWSI = 0.8 occurred when midday stem water potential was close to -1.5 MPa and stomatal conductance was lower than 200 mmol m^-2^s^-1^. Soil water content (SWC) was the first indicator in detecting the deficit irrigation applied, however, it was not as strongly related to the tree water status as the thermal-based indicators. Thus, the relation between the indicators studied with the stem water potential followed the order: CWSI > Tc-Ta > SWC = Tc. A multiple regression analysis is proposed that combines both soil water content and thermal-based indices to overcome limitations of individual use of each indicator.

## 1 Introduction

Precision agriculture uses information obtained from temporal or spatial data to address variability within the orchard, improve the productivity and quality of horticultural products, and enhance resource use efficiency and sustainability (Zhang et al., 2002). The application of sensors, remote sensing, information systems, and improved machinery are important for achieving low-input and high-efficiency horticulture (Zude-Sasse et al., 2021; Longchamps et al., 2022). Since irrigation is tightly associated with tree growth and productivity and water is an indispensable limited resource, technological tools that assess tree water status can improve irrigation scheduling and water use efficiency, avoiding over-irrigation or applying undesirable water stress (Gonzalez-Dugo et al., 2013; Blanco and Kalcsits, 2021).

According to the USDA, 2018 Irrigation and Water Management Survey, 22% of growers interviewed in the United States were using any type of sensor for irrigation management. Among all, the most common data-based method to schedule irrigation was soil sensors. Up to 65% more growers use soil sensors to schedule irrigation than those that use crop-water-evapotranspiration data. Even fewer growers use other methods, where soil sensors were used 5 and 14 times more than plant-based sensors or models, respectively (USDA, 2018). Based on this, the next step for growers is adding plant-based indicators to their soil sensors network that accurately provide low-cost and user-friendly information on the tree water status. There is a general agreement that the midday stem water potential is the reference tree water status indicator for fruit trees such as pear trees (*Pyrus communis* L.). However, its measurements are laborious (Marsal et al., 2000; Blanco et al., 2018). In the same vein, stomatal conductance has been widely reported as a sensitive physiological indicator of tree water status. However, it has not been implemented in commercial orchards because it is also labor intensive and requires highly technical and expensive equipment (Struthers et al., 2015; Noun et al., 2022). Other tree water status indicators such as canopy temperature (Tc) have also been identified (García-Tejero et al., 2011; Gonzalez-Dugo et al., 2012; Ramírez-Cuesta et al., 2022). Tc is strongly related to stomatal conductance by the direct relationship between high stomatal conductance, high transpiration rates, and the cooling effect of the vaporization of water. On the other hand, when stomatal conductance is lower from soil water deficits, transpiration decreases and canopy temperature increases. Despite this strong relationship with stomatal conductance, Tc is also highly affected by environmental conditions such as air temperature, vapor pressure deficit, and radiation (Mira-García et al., 2022). Thus, in order to be used as a tree water status indicator, Tc needs to be normalized to minimize its variation. To achieve that, several thermal indices have been proposed. Among these, the difference between canopy and air temperature (Tc-Ta) and the crop water stress index (CWSI) (Idso et al., 1981; Jackson et al., 1981) are those that have been widely reported as good water status indicators in many fruit trees such as citrus, nuts, and stone fruit (García-Tejero et al., 2011; Bellvert et al., 2018; Lee et al., 2019; Blaya-Ros et al., 2020).

Although pear trees are not drought resistant, previous research has highlighted the greater capacity to overcome water stress compared to other fruit trees such as apples or peaches. In this sense, deficit irrigation has proven to be a successful strategy in pears to control excessive vigor and increase flowering the following season (Marsal et al., 2012a). On the other hand, severe water stress will reduce pear fruit weight, yield, and relative gross revenue, with differences among cultivars, being ‘Blanquilla’/’Spadona di Salermo’/’Krystally’ more resistant to water stress than ‘Conference’ (Marsal et al., 2012a). Therefore, it is important to ensure water indicators that can accurately monitor tree water status and quantify the water stress applied to the trees to take full advantage of these irrigation strategies while avoiding the disadvantages that can cause.

Although new plant-based approaches that continuously assess tree water status have been recently developed (Lakso et al., 2022; Blanco and Kalcsits, 2023; Gonzalez Nieto et al., 2023), they are not as widely implemented in commercial fruit tree orchards as soil sensors, which are a major tool for precision irrigation of fruit trees (Vera et al., 2019). Thus, the combination of soil moisture sensors that continuously measure soil water status and plant-based punctual measurements can be an effective solution to assess tree water status and manage irrigation for commercial production. In this context, the development of low-cost, easy-to-use, portable infrared cameras can provide consistent and effective information on canopy temperature. Moreover, this technology can be incorporated into a smartphone giving growers the opportunity to rapidly include the use of image-based algorithms and thermal indices into their irrigation decision-making process (García-Tejero et al., 2018; Carrasco-Benavides et al., 2020).

The aim of this study was to compare traditional tree water status indicators (stem water potential and stomatal conductance) against the Tc-Ta and the CWSI obtained with thermal cameras for two different pear cultivars, ‘D’Anjou’ and ‘Bartlett’ (also known as ‘Williams’) under semi-arid climate conditions. We assessed if the combination of soil water content sensors and thermal imaging can provide robust information of the tree water status of pear trees.

## 2 Materials and methods

### 2.1 Experimental site and irrigation treatments

The experiment was conducted during two growing seasons, 2021 and 2022, in Rock Island (Washington State, USA, 47° 19′ N, 120° 04′ W) on a 0.81 ha orchard of two pear cultivars (*Pyrus communis* L.), ‘D’Anjou’ and ‘Bartlett’, grafted on OHxF.87 rootstock and trained to a central leader. The trees were planted in 2007 at a density of 833 trees ha^-1^. Full bloom occurred in April, and fruit was harvested in late August or early September.

Two irrigation treatments were imposed: A control treatment (CTL) that was irrigated at 100% of crop evapotranspiration (ET_C_) to fulfill tree water requirements and a regulated deficit irrigation treatment (DI), irrigated at 100% of ET_C_ from April 1^st^ to June 27^th^ in 2021 and April 1^st^ to June 24^th^ in 2022, and 50% of ET_C_ until the end of the season (October). ET_C_ was calculated using the methodology proposed by Allen et al. (1998):


$$ET_{C}=\;ET_{0}\;\times \;K_{C}\;\times \;K_{r}$$

where ET_0_ is the reference evapotranspiration, K_c_ is the crop-specific coefficient reported for adult pear trees (Marsal et al., 2012a), and K_r_ is a factor of localization (Fereres et al., 1981). Trees were drip irrigated by a system consisting of a single drip line per tree row and five emitters per tree of 2 L h^-1^ discharge rate.

Treatments were arranged in a completely randomized block design with three replicates per treatment and six trees per replicate. Two trees were selected because of the uniformity within each replicate at the beginning of the experiment for measurements. Horticultural practices followed commercial regular practices and were identical for both cultivars.

### 2.2 Environmental conditions and soil water content

Precipitation, solar radiation, and reference evapotranspiration were recorded by a weather station located at the orchard and owned by AgWeatherNet (http://www.weather.wsu.edu; “Sunrise station”). The air temperature and the relative humidity were recorded by two sensors (ATMOS-14, METER Group Inc., Pullman, WA, USA) installed in the pear orchard. Vapor pressure deficit (VPD) was calculated according to Allen et al. (1998). Soil volumetric water content (SWC) was obtained with two capacitance/frequency domain sensors (TEROS 11, Meter Group, Pullman, WA, USA) per replicate placed 25 and 50 cm below the surface and 25 cm from the drip emitter. The mean value of both sensors was calculated to estimate the soil volumetric water content at the root depth.

**Figure 1:**
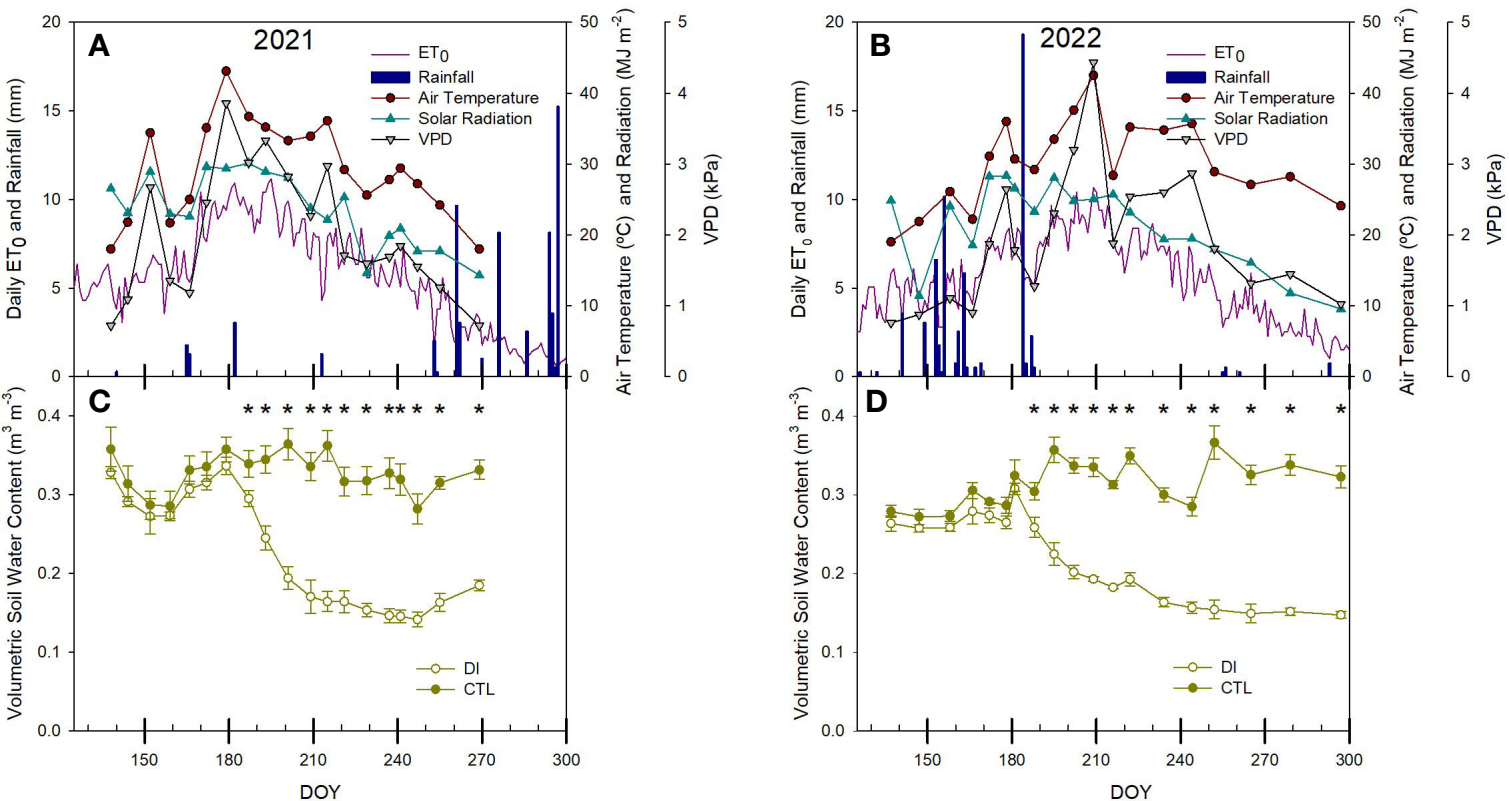
Seasonal environmental conditions **(A, B)** and soil water content **(C, D)** for the seasons 2021 and 2022 in the pear orchard. * denotes significant differences according to ANOVA (p< 0.05) between irrigation treatments.

Environmental conditions during the experimental period followed a similar pattern for both years, where both air temperature and evapotranspiration increased during spring from 18°C and 4 mm day ^- 1^ to reach maximum values of 43°C and 11 mm day^-1^ in the summer. However, the timing of the maximum summer temperatures was different each year. In 2021, the most demanding period of the season was earlier than in 2022, occurring in late June and early July (DOY 180 and 190) while in 2022, it occurred 30 days later, in late July (DOY 205-215). Moreover, the environmental conditions during late summer were also different. In 2021, the maximum air temperature and the ET_0_ rapidly decreased and several rainy episodes occurred from DOY 260 onwards. The maximum air temperatures in 2022 were nearly 27 °C until the end of the irrigated season (**Figure 1**).

### 2.3 Midday stem water potential and stomatal conductance

Midday stem water potential (SWP) was measured by using the Scholander pressure chamber (Model 615D, PMS Instrument Company, Albany, OR, USA) in two trees per replicate following the methodology described by McCutchan and Shackel (1992). Mature and healthy leaves close to the trunk were wrapped with black polyethylene bags and aluminum foil for two hours prior to measurement.

Stomatal conductance (Gs) was measured for two mature leaves from the outer part of the canopy per replicate, in the same trees in which the SWP was measured. Measurements were made at solar noon using a portable gas exchange system (LI-6400XT, Li-Cor Inc., Lincoln, NE, USA) equipped with a 2 cm^2^ chamber at CO_2_ concentration of 400 µmol CO_2_ mol^-1^ air. The airflow rate inside the chamber was set to 400 µmol s^−1^, 1500 μmol m^−2^ s^−1^ of photosynthetic photon flux density (PPFD) and leaf temperature was maintained at 25°C.

### 2.4 Canopy temperature and thermal-based indices

**Figure 2:**
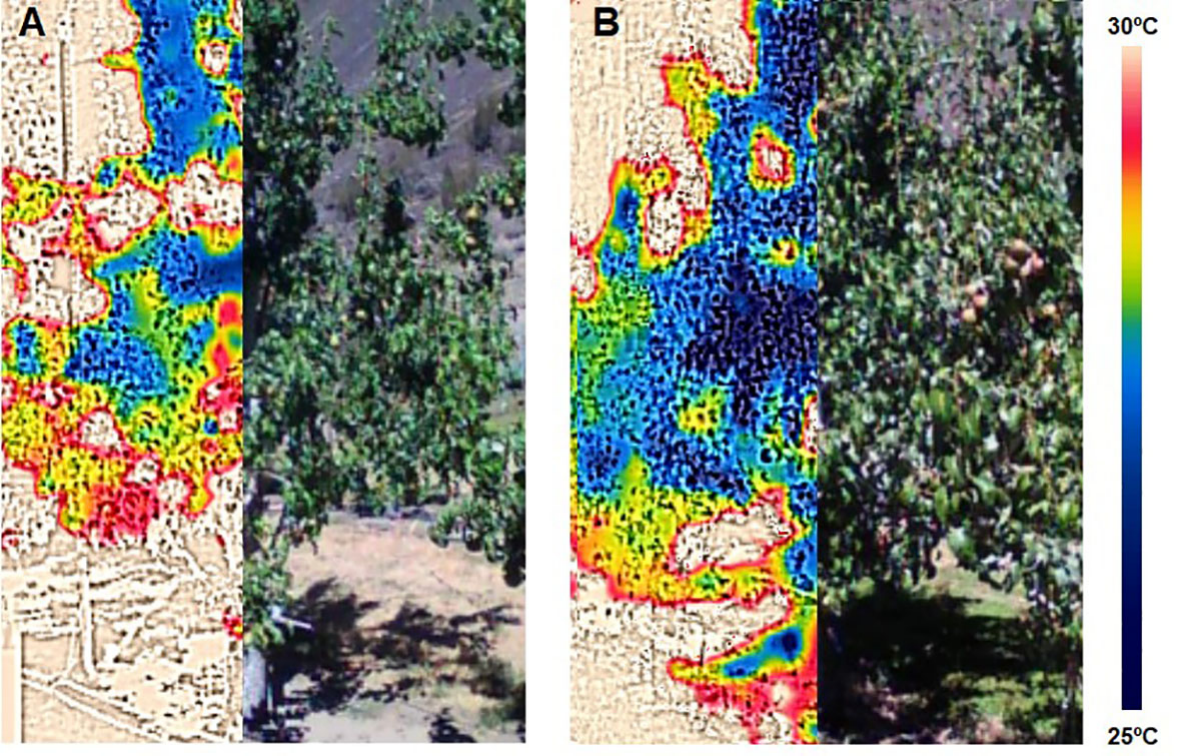
Images of the canopy and the canopy temperature of two pear trees, one under water deficit **(A)** and the other irrigated to satisfy its water needs **(B)** for the range 25 – 30°C, dark blue colors are canopy temperatures close to 25°C, yellow and red colors are canopy temperatures close to 30°C.

The canopy temperature (Tc) was measured at the same time and in the same trees as stomatal conductance and midday stem water potential, two trees per replicate, with a low-cost compact thermal camera (FLIR C2, FLIR Systems, Wilsonville, OR, USA). The camera uses a thermal sensor with a thermal resolution of 80 × 60 pixels, and ± 2% thermal accuracy. Images were taken 1.5 m from the sunny side of the canopy (**Figure 2**) and were analyzed using the FLIR Tools application (FLIR One, FLIR Systems, Wilsonville, OR, USA) according to Blaya-Ros et al., 2020.

Two thermal-based indices were calculated with the canopy temperature to diminish the effect of the environmental conditions and enhance effects of the irrigation treatments, the difference between the canopy and air temperature (Tc-Ta, Equation 1) and the crop water stress index (CWSI; Equation 2; Jackson et al., 1981).


(1) Tc−Ta  

(2) $$\text{CWSI}=\frac{\left(\text{Tc}-\text{Ta}\right)-\;\Delta {\text{T}}_{\text{wet}}}{\Delta {\text{T}}_{\text{dry}}-\Delta {\text{T}}_{\text{wet}}}\;$$

where Tc is the canopy temperature, Ta is the air temperature, ΔT_wet_ and ΔT_dry_ are the differences between canopy and air temperature when the leaves have the stomata fully transpiring and fully closed, respectively, for the specific environmental conditions of each day. ΔT_wet_ was calculated from non-water-stress baselines (NWSB) by spraying a thin layer of water on leaves 30 s before images were taken and ΔT_dry_ was calculated from non-transpiring baselines (NTB) by blocking all transpiration flows of the leaves by covering both sides of the leaves with a thin layer of Vaseline (Jones, 1999).

### 2.5 Statistical analysis

Data were analyzed by using analysis of variance (ANOVA) with a significance level of p< 0.05 and multiple regression analysis (IBM SPSS Statistics, SPSS Inc., 24.0 Statistical package, Chicago, IL, USA). Relationships between plant water status indicators were explored through linear and non-linear regression analyses performed with RStudio (RStudio Inc., Boston, MA, USA) and SigmaPlot 12.5 (Systat Software Inc., San Jose, CA, USA).

## 3 Results

### 3.1 Soil water content

Volumetric soil water content (SWC) was consistent between both irrigation strategies. SWC was steadier in the CTL treatment and ranged between 0.27 and 0.37 m^3^ m^-3^ across both years. SWC decreased in the DI treatment once deficit irrigation was imposed, decreasing from close to 0.32 m^3^ m^- 3^ to below 0.2 m^3^ m^-3^ at the end of each season, although the rate of decrease was different each year. SWC rapidly decreased in 2021 immediately after the water deficit was imposed. However, the decrease in SWC was slower in 2022 (**Figure 1**). As a result, significant differences between irrigation treatments were observed earlier in 2021 than in 2022 because the first days of water deficit coincided with the period of the highest evaporative demand (ET_0_ > 10 mm).

### 3.2 Midday stem water potential and stomatal conductance

**Figure 3:**
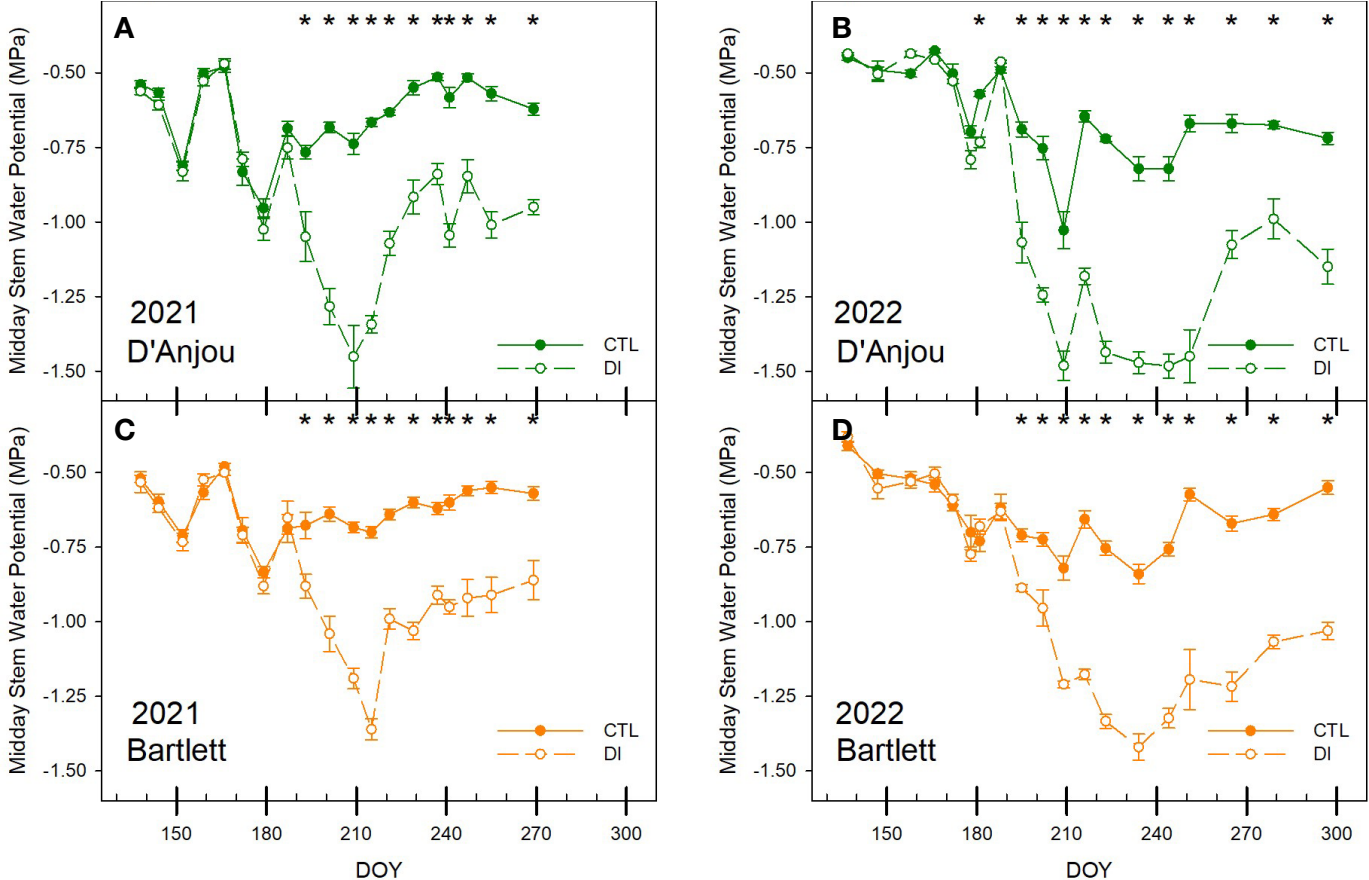
Seasonal pattern of midday stem water potential for ‘D’Anjou’ **(A, B)** and ‘Bartlett’ **(C, D)** for tree irrigated to satisfy their needs (CTL) and under water limitations (DI) in 2021 and 2022. * denotes significant differences according to ANOVA (p< 0.05) between irrigation treatments.

Soil water limitations affected tree water status for both cultivars. Midday stem water potential of DI trees was significantly lower than that of CTL trees once the deficit was imposed. Minimum stem water potential for DI trees were below -1.5 and -1.3 MPa in the summer for ‘D’Anjou’ and ‘Bartlett’ trees, respectively, for both years. However, lower summer stem water potentials persisted for longer during this period in 2022 than in 2021. Consequently, stem water potentials were lower in 2022 during the period from DOY 220 to 250 than those measured in 2021 (**Figure 3**).

**Figure 4:**
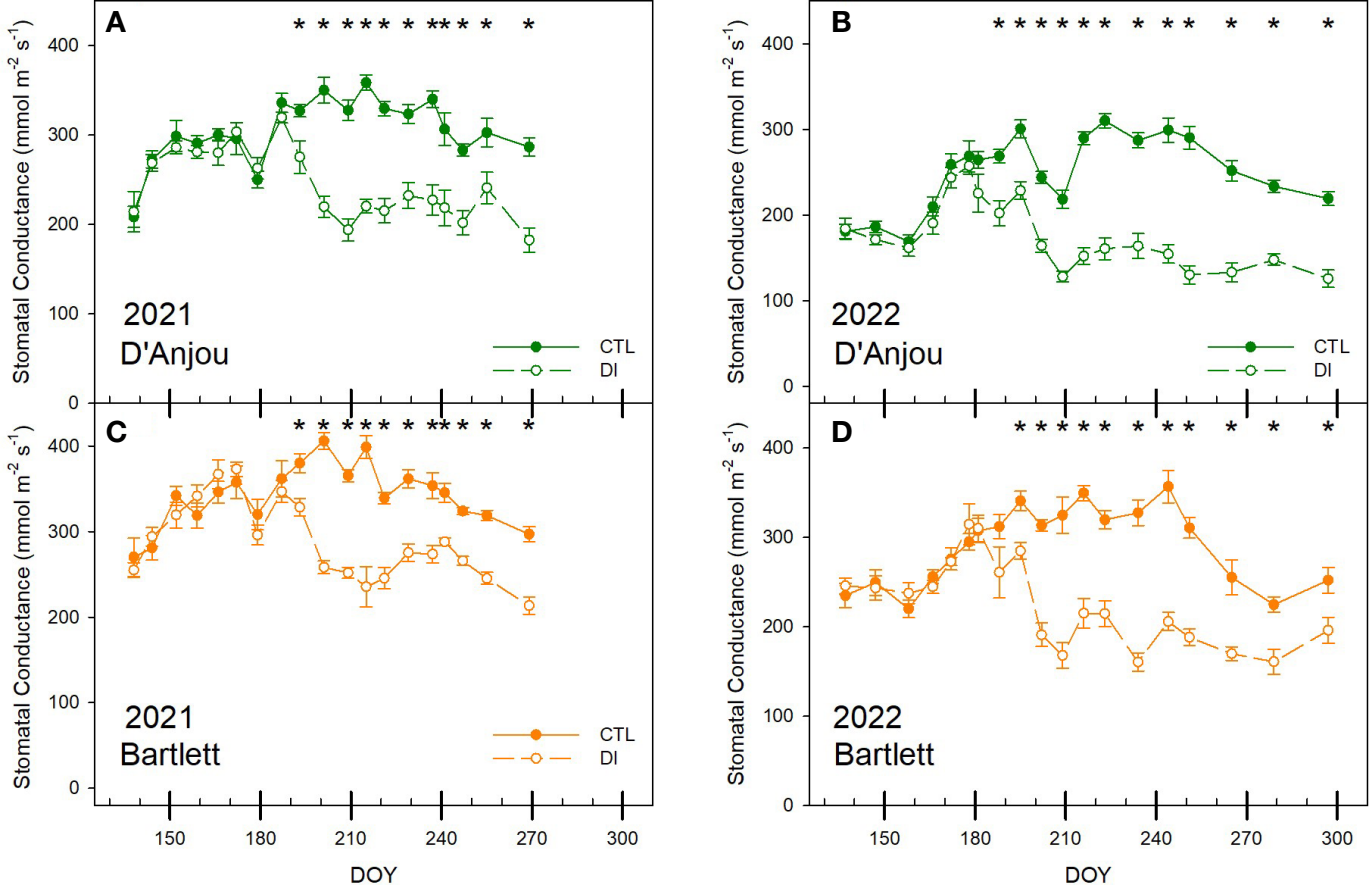
Seasonal patterns of stomatal conductance for ‘D’Anjou’ **(A, B)** and ‘Bartlett’ **(C, D)** for tree irrigated to satisfy their needs (CTL) or under water limitations (DI) in 2021 and 2022. * denotes significant differences according to ANOVA (p< 0.05) between irrigation treatments.

Similarly, when trees were not stressed, midday stem water potential was more stable for ‘Bartlett’ trees than for ‘D’Anjou’ trees with values ranging from -0.5 and -0.8 MPa and -0.5 and -1.0 MPa, respectively. Deficit irrigation significantly reduced stomatal conductance for both years and both cultivars. Stomatal conductance decreased by 37 and 28% in the first month after deficit irrigation was imposed for ‘D’Anjou’ and ‘Bartlett’ trees respectively. Differences between cultivars were more evident in 2022, when stomatal conductance for ‘D’Anjou’ under deficit irrigation became significantly lower than the control after 13 days while ‘Bartlett’ trees needed 19 days to show a significant reduction. When both years were compared, stomatal conductance was higher in 2021 than in 2022 for both treatments. Mean stomatal conductance for fully irrigated trees was 273 mmol m^-2^ s^-1^ in 2021 and 325 mmol m^-2^ s^-1^ in 2022 (**Figure 4**).

### 3.3 Canopy temperature and thermal-based indices

#### 3.3.1 Canopy temperature and the difference between canopy and air temperature

**Figure 5:**
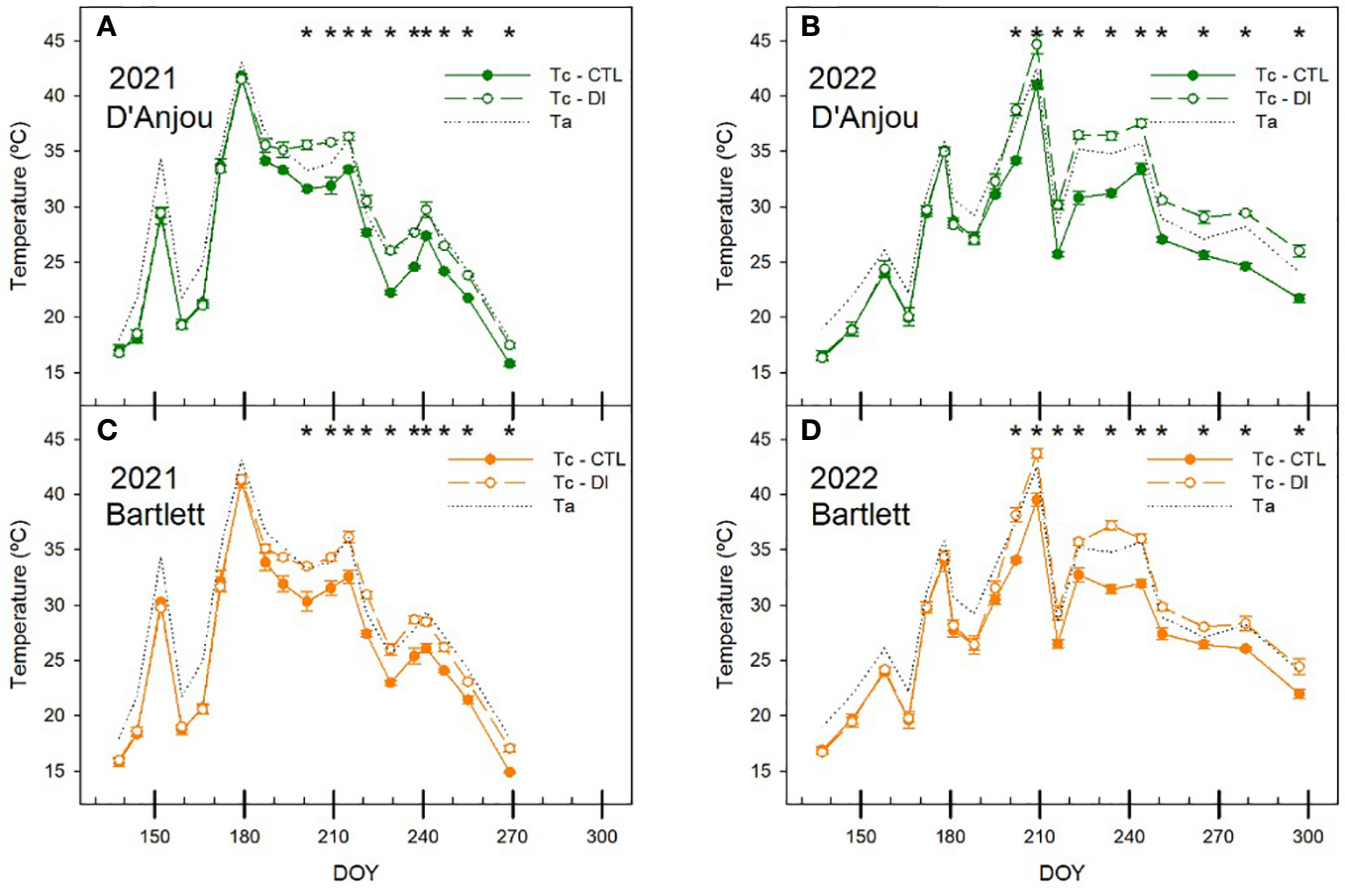
Seasonal patterns of air (Ta) and canopy (Tc) temperature for ‘D’Anjou’ **(A, B)** and ‘Bartlett’ **(C, D)** for trees irrigated to satisfy their needs (CTL) or under water limitations (DI) in 2021 and 2022. * denotes significant differences according to ANOVA (p< 0.05) between irrigation treatments.

The temperature of the canopy (Tc) was clearly influenced by stomatal conductance and environmental conditions (**Figure 5**). Tc was similar for both cultivars and significant differences between irrigation treatments occurred on the same dates. Maximum Tc occurred on days with the highest air temperature. The highest Tc (>44°C) during both seasons were measured in 2022 for DI trees and occurred on the same day when stem water potential was -1.5 MPa (DOY 209, **Figure 3**). Conversely, slightly lower Tc values were recorded for both CTL and DI trees in 2021 prior to the imposition of deficit irrigation when there were no soil water limitations and stem water potential for both cultivars was approximately -1.0 MPa (DOY 179).

**Figure 6:**
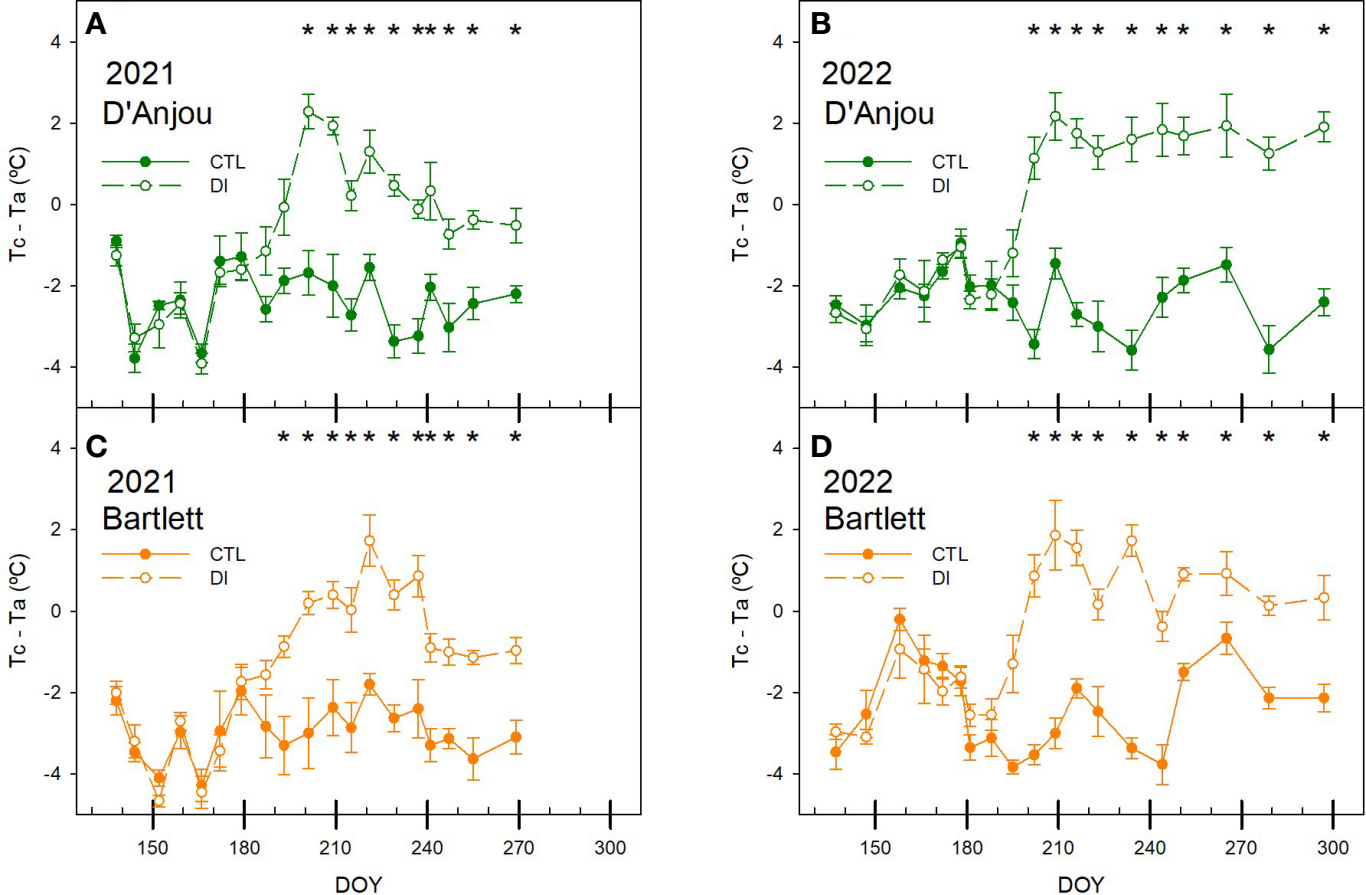
Seasonal patterns of the difference between the canopy and the air temperature (Tc-Ta) for ‘D’Anjou’ **(A, B)** and ‘Bartlett’ **(C, D)** for tree irrigated to satisfy their needs (CTL) or under water limitations (DI) in 2021 and 2022. * denotes significant differences according to ANOVA (p< 0.05) between irrigation treatments.

When Tc was compared to the air temperature (Ta), the effect of the environmental conditions was standardized and the difference between the two increased in response to stomata closure under deficit irrigation. When trees were irrigated to satisfy their water requirements, Tc-Ta was negative for both cultivars. However, when soil water limitations were applied, Tc-Ta became positive. This change of Tc-Ta from negative to positive values happened faster in 2021 than in 2022 and reached maximum values close to 2°C for both seasons. Similar to other water status indicators, in 2022 these maximum values were recorded several times from mid-summer onwards. The temperature of the canopy of the CTL trees was consistently 2.5°C below the air temperature for both cultivars. However, there were differences between cultivars under deficit irrigation. Although both cultivars had positive values of Tc-Ta under soil water deficits, ‘D’Anjou’ trees were 0.5 and 0.8°C higher than ‘Bartlett’. Thus, while ‘Bartlett’ never reached canopy temperature 2°C above the air temperature, Tc-Ta was greater than 2°C twice during the season 2021 and three times during the season 2022 for ‘D’Anjou’ trees under deficit irrigation. The maximum Tc-Ta reached (2.5°C) was observed on the day with the lowest stem water potential and stomatal conductance of the 2021 season (DOY 195) (**Figure 6**).

#### 3.3.2 Crop water stress index

**Figure 7:**
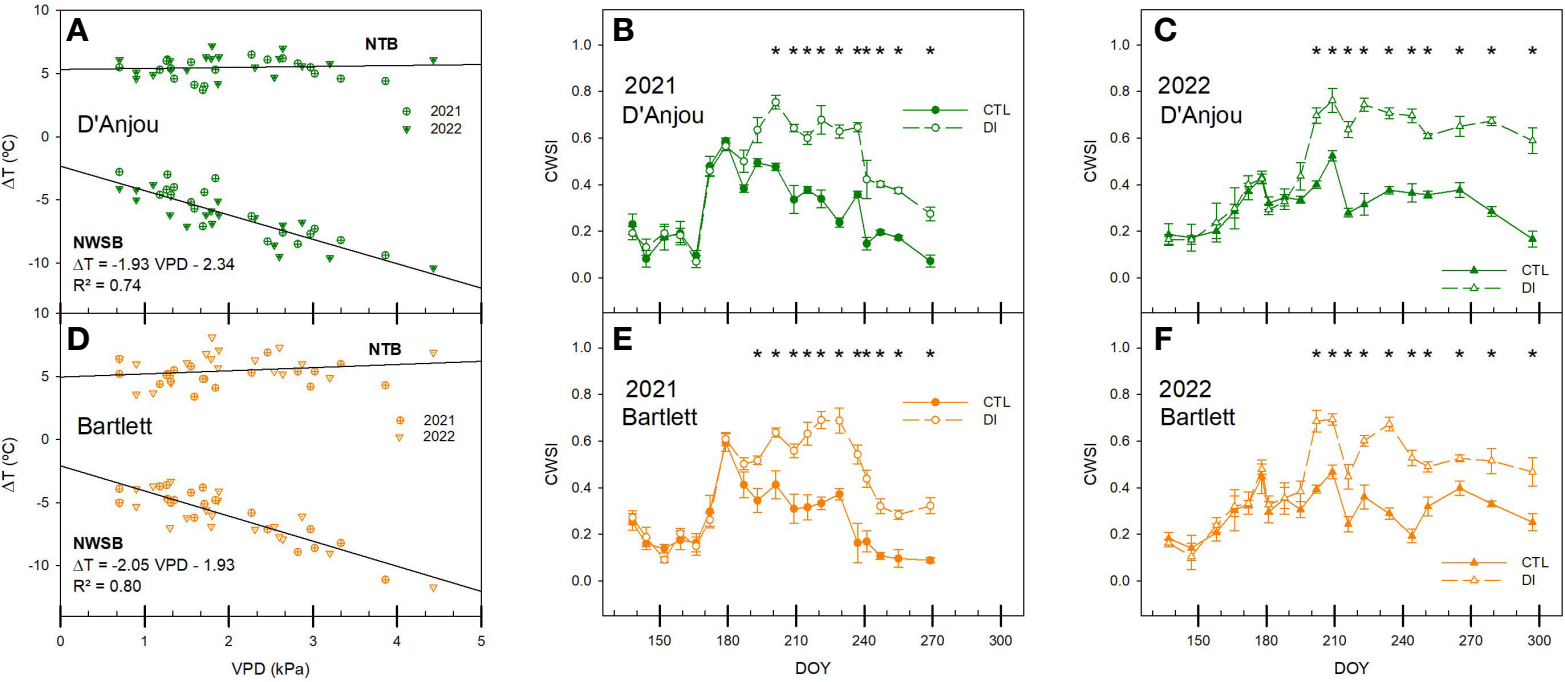
Graphical determination of the non-transpiring baselines (NTB) and non-water-stress baselines (NWSB) by comparing the vapor pressure deficit (VPD) and the difference between the air and canopy temperature (ΔT) and evolution of the crop water stress index (CWSI) for ‘D’Anjou’ **(A–C)** and ‘Bartlett’ **(D–F)** for tree irrigated to satisfy their needs (CTL) or under water limitations (DI) during the seasons 2021 and 2022. * denotes significant differences according to ANOVA (p< 0.05) between irrigation treatments.

With the values from both seasons, the non-water stress baseline (NWSB) and the non-transpiring baseline (NTB) were determined for both cultivars (**Figure 7**). The difference between the leaf temperature of non-water stressed leaves and the air temperature was clearly influenced by the VPD. Difference between the non-stressed leaves and the air increased as VPD increased. NWSB calculated for each cultivar was not significantly different, so when all data was pooled, the linear regression line obtained was: ΔT = – 1.99 VPD – 2.13 (R^2 = ^0.77). For non-transpiring leaves, both baselines were stable independent of environmental conditions and values recorded were slightly higher for ‘D’Anjou’ pear trees, with mean values of 5.3°C, than for ‘Bartlett’ trees (4.9°C).

Similar to the differences between canopy and air temperature, CWSI responded to deficit irrigation imposed on DI trees. Moreover, CWSI significantly differed between both irrigation treatments earlier in ‘Bartlett’ trees than in ‘D’Anjou’ in 2021 due to a lower variability among trees observed for ‘Bartlett’. This difference between cultivars was not present in 2022. CWSI was between 0.1 and 0.5 for CTL trees. However, when the air temperature reached 45°C in 2021, the CWSI of CTL trees was 0.6, despite having values of soil water content close to field capacity (> 0.3 m^3^ m^-3^, **Figure 1**). Although similar air temperatures were recorded in mid-summer of 2022, the CWSI of CTL trees never exceeded the threshold value of 0.5. DI trees showed CWSI values consistently higher than 0.6 for ‘D’Anjou’ trees and 0.5 for ‘Bartlett’ trees, but always lower than 0.8 (**Figure 7**).

### 3.4 Relationships between soil and plant water status indicators and thermal-based indices

**Figure 8:**
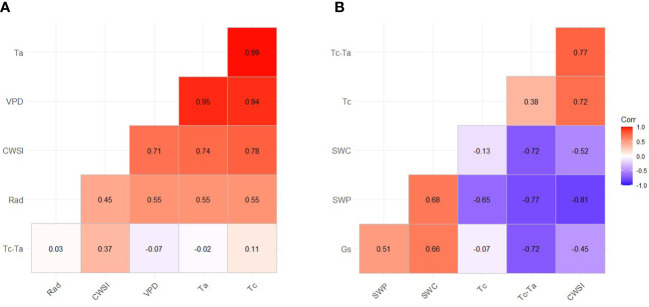
Correlation matrix (and correlation coefficients) for the thermal-based indices and the environmental conditions **(A)** or the soil and plant water status indicators **(B)**. Ta, Air temperature; Rad, Solar radiation; VPD, Vapor pressure deficit; CWSI, Crop Water Stress Index; Tc-Ta, Difference between the temperature of the canopy and the air; Tc, Temperature of the canopy; SWC, Soil water content; SWP, Midday stem water potential; Gs, Stomatal conductance.

All data were pooled to calculate two correlation matrices with environmental conditions and soil/plant water status indicators studied (**Figure 8**). As expected, Tc resulted strongly related to the air temperature and VPD, while Tc-Ta was not as related. Regarding the water status indicators, for the midday stem water potential, in order of relevance, both the CWSI and the difference between the canopy and air temperature were strongly coupled with it. Soil water content and canopy temperature were also able to explain variability in stem water potential. Stomatal conductance was the least correlated with stem water potential (r = 0.51). When the stomatal conductance was compared with other water status indicators and indices, Tc-Ta was the thermal index with the strongest relationship. Surprisingly, no association was found between the canopy temperature and stomatal conductance, highlighting the effect of environmental conditions on canopy temperature compared to the effect of water deficit. Importantly, soil water content explained 46 and 44% of stem water potential and the stomatal conductance variation, respectively.

**Figure 9:**
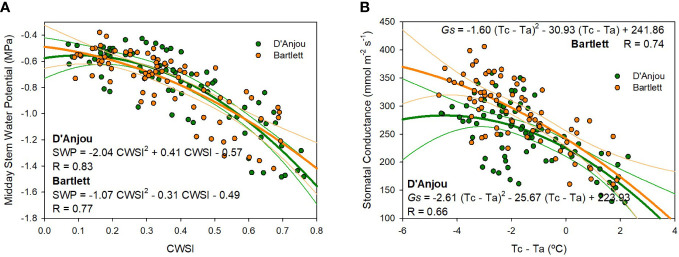
Non-linear relationship between the crop water stress index (CWSI) and the midday stem water potential **(A)** and the relationship between the canopy and air temperature (Tc-Ta) and the stomatal conductance **(B)** for ‘D’Anjou’ and ‘Bartlett’ pear trees in 2021 and 2022.

There was a second-degree polynomial relationship between midday stem water potential, stomatal conductance, and the thermal indices calculated for each cultivar (**Figure 9**). The relationship between stem water potential and CWSI behaves similarly for both ‘D’Anjou’ and ‘Bartlett’. According to these relationships, values of CWSI higher than 0.45 indicate trees are under water deficit and values higher than 0.7 indicate trees are under severe water stress. Conversely, differences between the two cultivars were found when assessing stomatal conductance. Under no water restrictions, ‘Bartlett’ pear trees had higher stomatal conductance values compared to ‘D’Anjou’ trees. However, similar results for both cultivars were observed when the trees were under water restrictions, with values lower than 220 mmol m^-2^ s^-1^ when the canopy temperature was higher than the air temperature (Tc- Ta > 0; **Figure 9**).

**Table 1** shows a multiple regression analysis in which stem water potential and stomatal conductance were estimated as a function of soil water content and thermal indices. For stem water potential, CWSI, the difference between the canopy and air temperatures and soil water content improved the estimates compared with the soil water content alone. On the other hand, for stomatal conductance, the combination of the soil water content with the thermal indices did not improve the explanation of variance if only the Tc-Ta was considered.

**Table 1:** Multiple regression analysis to predict midday stem water potential and stomatal conductance from CWSI (crop water stress index), SWC (soil water content), and Tc-Ta (difference between air and canopy temperature).

R		Coefficients	Standard Error	Signific	Confidence Interval	
					Lower limit	Upper limit
Midday stem water potential						
0.862	Independent term	-0.841	0.070	<0.001	-0.981	-0.702
	CWSI	-0.941	0.071	<0.001	-1.080	-0.801
	SWC	1.513	0.194	<0.001	1.130	1.896
0.796	Independent term	-1.235	0.070	<0.001	-1.374	-1.096
	Tc-Ta	-0.088	0.011	<0.001	-0.111	-0.066
	SWC	1.152	0.297	<0.001	0.565	1.740
Stomatal conductance						
0.669	Independent term	134.019	23.950	<0.001	86.694	181.345
	CWSI	-45.871	24.014	<0.001	-93.322	1.580
	SWC	554.271	65.892	<0.001	424.067	684.474
0.723	Independent term	152.201	17.827	<0.001	116.976	187.427
	Tc-Ta	-15.080	2.859	<0.001	-20.730	-9.430
	SWC	334.698	75.363	<0.001	185.780	483.615

## 4 Discussion

In pear trees, controlling the excessive vegetative growth is required (Marsal et al., 2002; Carra et al., 2017). Irrigation strategies such as regulated deficit irrigation have shown benefits for many fruit tree species, reducing vegetative growth while increasing fruit quality (Galindo et al., 2018; Martínez-Hernández et al., 2020; Reid and Kalcsits, 2020). These strategies have been successfully applied in pear trees under different environmental conditions (Mitchell et al., 1989; Marsal et al., 2012b; Moreno-Hernández et al., 2017; Venturi et al., 2021). In order to implement these strategies, the impact of the water deficit on physiological responses needs to be quantified.

Midday stem water potential and stomatal conductance have been traditionally used to assess tree water status due to their robust and fast response to soil water limitations (Naor 2000; Van Beek et al., 2013; Conesa et al., 2019). Many studies have reported strong relationships between thermal-based indices, stem water potential and stomatal conductance (García-Tejero et al., 2018; Ahumada-Orellana et al., 2019; Park et al., 2021). Our results are aligned with those studies and show high correlation coefficients between thermal-based indices and traditional water status indicators (**Figure 8B**). Similar correlation coefficients to those obtained for pear trees (r = -0.65) were also reported in olive, citrus, almond, and walnut trees (Ben-Gal et al., 2009; Stagno et al., 2011; Dhillon et al., 2014). The findings reported in this study highlight the usefulness of thermal indices for assessing the tree water status of pear trees.

In the case of stomatal conductance, although deficit irrigation led to lower values and higher canopy temperatures, there was no relationship between the two due to the high influence of air temperature on canopy temperature. Low correlation coefficients between stomatal conductance and Tc were also reported in sweet cherry trees by Carrasco-Benavides et al. (2020) using a similar low-resolution thermal infrared camera, indicating limitations of Tc as a tree water status indicator. As such, it was not possible to identify a threshold for identifying water deficits (**Figure 5**) because of the high dependency of tree canopy temperature in relation to air temperature and tree phenological stage (**Figure 8**; Leuzinger and Körner, 2007; Kim et al., 2016). Our results stress the use of the Tc-Ta index over absolute values of Tc. Stomatal conductance and midday stem water potential were strongly related to Tc-Ta for this study. Tc-Ta has been reported to be strongly related to stem water potential in other woody crops such as stone fruits, citrus, nuts, and vines (Wang and Gartung, 2010; Gonzalez-Dugo et al., 2012; Bellvert et al., 2014; Gonzalez-Dugo et al., 2014).

The daily pattern of the canopy temperature and its relationship with stem water potential in ‘D’Anjou’ pear trees was also observed in a previous study (Blanco and Kalcsits, 2021). In pear trees, values of Tc-Ta similar to 2°C were measured during the experiment for DI trees under water restrictions with stem water potential values that ranged from -1.2 to -1.5 MPa. Slightly lower values of Tc-Ta (1.5°C) were reported by Blaya-Ros et al. (2020) for mature sweet cherry trees under similar soil water restrictions and midday stem water potentials of -1.2 MPa, but values of Tc-Ta higher than 4°C were reported in peach trees under deficit irrigation when stem water potential dropped below -2.0 MPa (Wang and Gartung, 2010). Based on these results, 1.5°C might be the threshold value of Tc-Ta proposed for pear trees in order to activate the irrigation as has been reported for ‘Bartlett’ that minimum values of midday stem water potential below -1.4 MPa during the second stage of fruit growth decrease fruit size (Marsal et al., 2002). Tc-Ta for CTL trees were always below 0°C and averaged -2°C, even under maximum air temperatures higher than 40°C (**Figure 6**). Negative values of Tc-Ta are associated with trees under no soil water content restrictions (Bellvert et al., 2014). Thermal-based sensors have also been reported to be more reliable than stem water potential for assessing tree water status in soils at field capacity (Conesa et al., 2023). Considering that over-irrigated pear trees that have excessive vigor lead to reduced fruit yield, a range of Tc-Ta between -2 and 1.5°C is proposed as the ideal range to avoid penalties for severe water deficit or overirrigation.

When Tc-Ta was compared with the VPD, to calculate the NTB and NWSB, NTB was similar to that proposed by Jones et al. (2018) at 5°C, and higher than that stated by Gençoğlan and Gençoğlan (2018) in ‘Comice’ pear trees at 3 – 4°C. For the NWSB, our results were obtained under a wider range of VPD values, between 0.7 and 4.5 kPa, compared to the lower limit of non-water stressed trees calculated for ‘Comice’. When both baselines were compared at VPD values of 2 kPa, Tc-Ta values were approximately -7°C. However, for lower VPD values, the limits proposed for ‘D’Anjou’ and ‘Bartlett’ trees were more negative than those proposed to ‘Comice’ trees. The values of CWSI calculated in this study were similar to those reported by Egea et al. (2017) in olive trees under severe water stress but lower than those reported in apple trees under no water stress by Mohamed et al. (2021). The difference in the values reported by Mohamed et al. (2021) under similar environmental conditions might be attributed to the calculation of the NWSB, as that study used an approach based on environmental conditions which might overestimate their calculation of the CWSI compared to when non-stressed leaves are directly measured. In our study, CWSI for CTL trees ranged between 0.1 and 0.4, although they increased to 0.5 during the heat dome event of 2021, indicating values of CWSI similar to 0.5 can be measured when environmental conditions are highly demanding. For DI trees, CWSI reached values of 0.8 when the midday stem water potential was as low as -1.5 MPa and the stomatal conductance dropped below 200 mmol m^- 2^s^- 1^. Values of CWSI similar to 0.55 have been reported by Gençoğlan and Gençoğlan (2018) in ‘Comice’ pear trees irrigated to satisfy their water needs while the CWSI was similar to 0.8 for pear trees under water deficit. These results suggest a similar pattern for pear trees when combined with our study. Although the response to water deficit was not the same for both pear cultivars, values of CWSI higher than 0.5 were always related to trees under water stress conditions.

‘Bartlett’ pear trees were less stressed than ‘D’Anjou’ trees under the same soil and weather conditions, potentially due to differences in the tree’s vegetative growth. Policarpo et al. (2006) reported that ‘Bartlett’ pear trees tend to have bigger canopies compared to other pear cultivars, which might cause higher transpiration and, consequently, lower canopy temperature compared to ‘D’Anjou’ trees under the same soil water restrictions and environmental conditions. In this sense, CWSI seemed to be more sensitive to identifying water deficit in ‘Bartlett’ trees than in ‘D’Anjou’ trees. These differences between cultivars were also observed in sweet cherry trees, where CWSI was more sensitive for identifying water deficit in the cultivar with the highest stomatal conductance (Carrasco-Benavides et al., 2020). The results reported in the present work show the potential of using CWSI and Tc-Ta for assessing the water status of pear trees and provide threshold values related to the physiological response of the tree. This represents an advancement in the implementation of the use of thermal-based indicators which, jointly with the development of low-cost, easy to interpret continuous sensors, can be useful for irrigation scheduling (Giménez-Gallego et al., 2023).

Soil moisture sensors are still extensively used for making irrigation decisions. This may be due to their sensitivity to water limitations. Although several studies have reported high variability when measuring soil moisture under field conditions compared to other plant-based indicators (Blanco et al., 2018; Domínguez-Niño et al., 2020), in our experiment soil water content detected earlier water deficit than thermal-based indicators. Nine and 15 days after the deficit irrigation was imposed on DI trees in 2021 and 2022 respectively, soil water content was lower for DI treatments compared to the CTL. However, on the same date, there were no differences between irrigation treatments when thermal-based indices were used. These results agree with those described by Mira-García et al. (2022) who reported that thermal indices were not able to detect water stress in lime trees earlier than soil water content. In our study, soil water content was the first water status indicator that detected significant differences between irrigation treatments, followed 7 days later by the midday stem water potential and the stomatal conductance, and 14 days later by the thermal-based indices. Similarly, Struthers et al. (2015) observed in young pear potted trees from the cultivar ‘Conference’ that Tc of water-stressed trees showed significant differences compared with the well-watered trees after 18 days of water stress while other indicators such as stomatal conductance showed significant differences between irrigation treatments after nine days. This can be explained by the physiological response of both pear cultivars to slight/early water deficits, since stomata remain open and maintain high transpiration rates during the first days of water restrictions which consequently, did not lead to increased Tc.

The multiple linear regression presented in this study shows the combination of soil water content and Tc-Ta can be useful for assessing tree water status, particularly to estimate midday stem water potential. Soil water content and environmental conditions have been successfully used in fruit trees to estimate tree water status (Martí et al., 2013; González-Teruel et al., 2022). We propose the use of Tc-Ta compared with CWSI because determination coefficients calculated were similar for both indices and Tc-Ta does not require the calculation of the upper and lower limit baselines. Similarly, although the results of the non-linear regression relationship of CWSI, Tc-Ta and midday stem water potential and the stomatal conductance (**Figure 9**) showed similar results to those of the multiple linear regression analysis, we propose the use of the combination of Tc-Ta and soil water content, since Tc-Ta alone was not precise enough to detect slight water deficits.

## 5 Conclusions

The results presented in this study show that the combination of Tc-Ta and soil water content sensors provides accurate information on tree water status and overcomes the limitations of their individual use. Thus, soil water content sensors detected water deficit earlier than thermal-based indices. However, when they were compared to midday stem water potential, they were not as strongly related compared to thermal-based indices. On the other hand, thermal-based indices were more closely related to tree water status during the season even though it took longer for irrigation treatments to influence this measured variable. The non-limiting baseline for each pear cultivar proposed in this work applied to a wide range of VPD conditions. This stable baseline may help facilitate the use of the CWSI by pear growers for irrigation scheduling. CWSI values higher than 0.7 were always related to severe water stress. The use of the Tc is not recommended, particularly to estimate stomatal conductance. Although Tc increased as a response to the stomata closure, environmental conditions had a greater impact. Measures that best integrate tree water status, soil water availability and atmospheric demand at a physiological scale will continue to be the best indicators of water stress to make precise irrigation decisions in pears.

## Data availability statement

The raw data supporting the conclusions of this article will be made available by the authors, without undue reservation.

## Author contributions

Conceptualization and design, VB and LK. Methodology, VB, NW, TC, OH, and LK. Investigation, VB, NW, TC, OH, and LK. Writing—original draft preparation, VB, NW, TC, OH, and LK. Writing—review and editing, VB, NW, TC, OH, and LK. Visualization, VB and LK. Supervision, VB and LK. Project administration and funding acquisition, VB and LK. All authors contributed to the article and approved the submitted version.
